# Kinetic, Equilibrium and Thermodynamic Studies of Cadmium (II) Adsorption by Modified Agricultural Wastes

**DOI:** 10.3390/molecules161210443

**Published:** 2011-12-15

**Authors:** Zeid A. Al Othman, Ali Hashem, Mohamed A. Habila

**Affiliations:** 1 Department of Chemistry, Collage of Science, King Saud University, Riyadh 11451, Saudi Arabia; 2 National Research Centre, Dokki, Cairo 12622, Egypt

**Keywords:** *Tamrix articulata* wastes, cadmium removal, adsorption, kinetic and thermodynamic study

## Abstract

Agricultural wastes have great potential for the removal of heavy metal ions from aqueous solution. The contamination of water by toxic heavy metals is a worldwide environmental problem. Unlike organic pollutants, the majority of which are susceptible to biological degradation, heavy metals do not degrade into harmless end products. Discharges containing cadmium, in particular, are strictly controlled because of the highly toxic nature of this element and its tendency to accumulate in the tissues of living organisms. This work aims to develop inexpensive, highly available, effective metal ion adsorbents from natural wastes as alternatives to existing commercial adsorbents. In particular, *Tamrix articulata* wastes were modified chemically by esterification with maleic acid to yield a carboxyl-rich adsorbent. The adsorption behavior of treated *Tamrix articulata* wastes toward cadmium ions in aqueous solutions in a batch system has been studied as a function of equilibration time, adsorbent dose, temperature and pH. Results showed that the maximum adsorption capacity was 195.5 mg/g in a pH 4 solution at 30 °C with a contact time of 120 min, an initial concentration of 400 mg/L and an adsorbent dose of 0.3 g/L. The kinetic data were analyzed using pseudo-first-order and pseudo-second-order kinetic models. It was shown that the adsorption of cadmium could be described by a pseudo-second-order equation. The experimental data were also analyzed using the Langmuir and Freundlich models of adsorption. Thermodynamic parameters such as ΔG^o^, ΔH^o^ and ΔS^o^ have been evaluated and it has been found that the sorption process was spontaneous and exothermic in nature. From all of our data, we conclude that the treated *Tamrix* *articulata* wastes investigated in this study showed good potential for cadmium removal from aqueous solutions.

## 1. Introduction

Cadmium (Cd) is a toxic heavy metal of significant environmental and occupational concern [1]. It is introduced into water from smelting, metal plating, cadmium nickel batteries, phosphate fertilizers, mining, pigments, stabilizers, alloy industries and sewage sludge [2]. Contamination of drinking-water may occur as a result of the presence of cadmium as an impurity in the zinc of galvanized pipes or cadmium-containing solders in fittings, water heaters, water coolers and taps. In Saudi Arabia, mean concentrations of 1–26 µg/L were found in samples of potable water, some of which were taken from private wells or cold corroded pipes [3]. Cadmium has been classified as a human carcinogen and teratogen, impacting the lungs, kidneys, liver and reproductive organs [1,4]. The harmful effects of cadmium include a number of acute and chronic disorders, such as renal damage, emphysema, hypertension and testicular atrophy [2]. Hence, it is essential to remove Cd (II) from water and wastewater prior to transport to prevent cycling into the natural environment. The most important technologies employed to remove cadmium include chemical precipitation, electroflotation, ion exchange, reverse osmosis and adsorption onto activated carbon [5]. Adsorption has been developed as an efficient method for the removal of heavy metals from contaminated water and soil. A variety of adsorbents, including clays, zeolites, dried plant parts, agricultural waste biomass, biopolymers, metal oxides, microorganisms, sewage sludge, ash and activated carbon have been used for cadmium removal [6,7,8,9,10,11,12,13,14,15,16,17,18,19,20,21,22]. Cost is an important parameter for comparing adsorbent materials [23]. Activated carbon is considered to be a highly effective adsorbent for heavy metal removal from wastewater, but it is readily solubilized under extreme pH conditions [24] and is also very high cost [25]. Low-cost agricultural waste byproducts, such as sugarcane bagasse [26], rice husks [27], sawdust [28], coconut husks [29], oil palm shell [30], and neem bark [31], have been investigated to discover if they can eliminate heavy metals from wastewater. In this study, *Tamrix* *articulata* wastes were modified chemically by esterification with maleic acid under conditions that yielded a carboxyl-rich adsorbent. The treated adsorbent was used to remove cadmium (II) ions from water. The equilibria, kinetics and thermodynamics of the sorption process were examined.

## 2. Results and Discussion

### 2.1. Properties of Native and Maleic Acid-Treated Tamrix articulata Wastes

**Figure 1:**
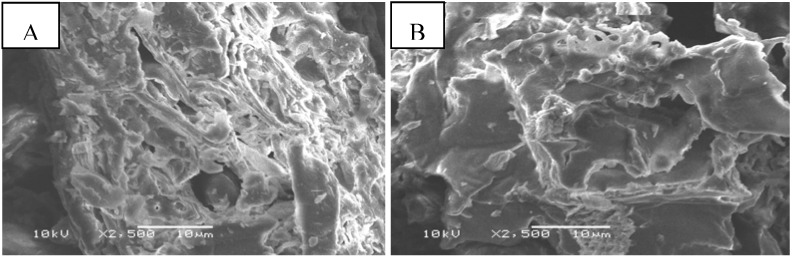
Shows scanning electron microscope (SEM) images of native waste surfaces (**A**) and maleic acid-treated *Tamrix articulata* wastes (**B**).

FTIR data showed bands at 3,640–3,500 cm^−1^, corresponding to O–H stretching vibrations, 3,000–2,800 cm^−1^ which correspond to C–H stretching vibrations, and 1,740–1,725 cm^−1^ which correspond to COO and C–O vibrations, respectively. Further bands at 1,130–1,000 cm^−1^ correspond to the C–O–C and O–H vibrations of polysaccharides. The same peaks were observed for native and maleic acid-treated *Tamrix articulata* wastes. The only difference observed was an increase in the intensity of the peak at 1,735 cm^−1^ in the case of treated *Tamrix articulata* wastes, which confirms the increase in COO groups due to esterification. The elemental analysis results for native and maleic acid-treated *Tamrix articulata* wastes revealed that native wastes are composed of 41.7% carbon, 5.3% hydrogen, and 1.8% nitrogen, whereas maleic acid-treated *Tamrix articulata* wastes are composed of 45.4% carbon, 5.6% hydrogen, and 1.0% nitrogen. The increase in the carbon content is due to the increase in carboxyl content. Figure 1 shows scanning electron microscope (SEM) images that reveal the rough and microporous structure of the surfaces of native (Figure 1A) and maleic acid-treated *Tamrix articulata* wastes (Figure 1B). Esterification appears to have only resulted in changes in the chemistry of the surface and did not clearly change the porosity of the sample.

### 2.2. Effect of Carboxyl Group Content of Treated Tamrix articulata Wastes on Cd (II) Adsorption

Table 1 shows the effect of the carboxyl group content of the esterified *Tamrix articulata* wastes on their adsorption capacity (metal uptake per unit weight of adsorbent) for Cd (II) ions from aqueous solutions. Evidently, increasing the extent of esterification was accompanied by an increase in the adsorption capacity of the treated *Tamrix articulata* wastes. A greater extent of esterification is accompanied by formation of the cellulose maleate (Equation 1), which increases the adsorption of Cd (II) ions. The data show that as the concentration of maleic acid increased from 0.0005 to 0.0043 mmol/g *Tamrix articulata* waste, the extent of esterification increased from 131.2 to 399 mg equiv COOH/100 g sample, with a corresponding increase in the adsorption capacity from 53.3 to 195.5 mg/g. These results indicate that the carboxyl groups introduced into the cellulose structure of *Tamrix articulata* wastes via esterification, play an important role in the Cd (II) ion adsorption process.

**Table 1:** Effect of esterification of *Tamrix articulata* wastes on Cd (II) ion adsorption at 30 °C.

Maleic Acid (mmol/g *waste)*	Esterification (Carboxylic group content) (mequiv/100 g Sample)	Adsorption Capacity (qe) (mg/g)
0.0043	399	195.5
0.0022	315	137.70
0.0011	210	88.84
0.0005	131.2	53.3

### 2.3. Adsorption Studies

#### 2.3.1. Effect of pH

**Figure 2:**
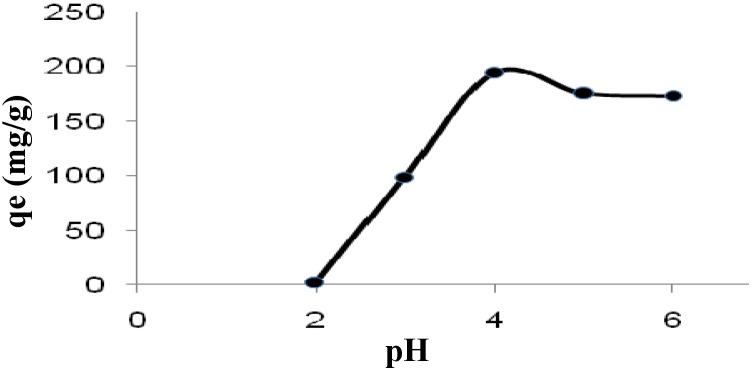
Effect of pH on cadmium biosorption by modified *Tamrix articulata* wastes at 30 °C.

As shown in Figure 2, the adsorption capacity (mg/g) of modified *Tamrix articulata* wastes for Cd (II) ions increased over the range 2.6–194.5 mg/g as pH values increased from 2 to 4. Indeed, the maximum adsorption, q_max_, of Cd (II) ions was attained at a pH value of 4. This could be ascribed to the lower stability of chelates formed in highly acidic media. At lower pH values, the H ions present in the system compete with metal cations for the exchange sites on the adsorbent surface, causing partial release of these cations. Heavy metal cations are completely released under extremely acidic conditions [32].

#### 2.3.2. Kinetics of Adsorption

**Figure 3:**
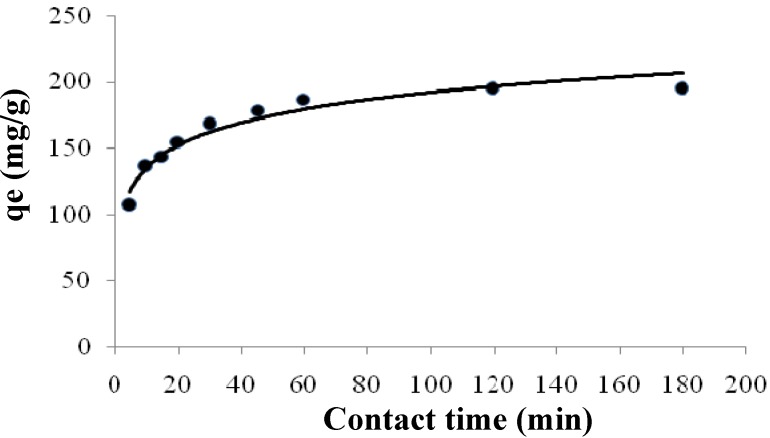
Effect of contact time on cadmium (II) biosorption by modified *Tamrix articulata* wastes at pH 4 and 30 °C.

Information on the kinetics of solute uptake is required to select optimal operating conditions for full-scale batch processing. The graph plotted in Figure 3 shows that a contact time of 120 min was sufficient to achieve equilibrium and that the adsorption did not change significantly with further increases in the contact time. Therefore, the uptake and un-adsorbed cadmium concentrations at the end of 120 min are given as the equilibrium values.

The kinetic values of adsorption were analyzed using pseudo-first-order and pseudo-second-order kinetic models. These models correlate solute uptake to predict the required reactor volume. These models are explained further below. The pseudo-first-order equation of Lagergren [33] is generally expressed as in Equation (1):

*dq_t_/dt = k_1_(q_e_ − q_t_)* (1)

where q_e_ and q_t_ are the sorption capacities at equilibrium and at time t, respectively, and k_1_ is the rate constant of pseudo-first-order sorption (min^−1^). After integration and applying boundary conditions, q_t_ = 0 to q_t_ = q_t_ at t = 0 to t = t, the integrated form of Equation (1) becomes:

*log(q_e_−q_t_) = log q_e_−k_1_t/2.303* (2)

**Figure 4:**
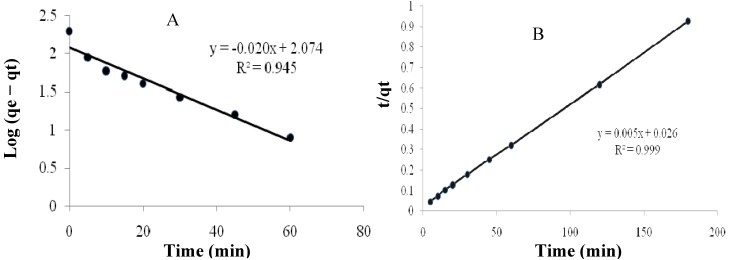
Pseudo-first-order (A) and pseudo-second-order (B) cadmium (II) biosorption by modified *Tamrix articulata* wastes.

The pseudo-first-order rate constant, k_1_, can be obtained from the slope of the graph of log(qe-q)*versus* time t (Figure 4A). The calculated k_1_ values and corresponding linear regression correlation coefficient values are shown in Table 2. The linear regression correlation coefficient value R_1_^2^ was 0.945, which indicates that this model cannot be applied to predict the adsorption kinetics.

**Table 2:** Kinetic constants for cadmium(II) adsorption onto maleic acid-treated *Tamrix articulata* wastes at an initial concentration of 400 mg/L at 30 °C and pH 4.

The pseudo-first-order			The pseudo-second-order		
**Rate Constant (K_1_)**	**qe (mg/g)**	**(R_1_^2^)**	**Rate Constant (K_2_)**	**qe (mg/g)**	**(R_2_^2^)**
0.046	118.5	0.945	0.00096	200	0.999

The pseudo-second-order chemisorption kinetic rate equation is expressed in Equation (3) [34]:

*dq_t_/dt = k_2_(q_e_ − q_t_)^2^* (3)

where q_e_ and q_t _are the sorption capacities at equilibrium and at time t, respectively, and k_2_ is the rate constant of pseudo-second-order sorption (g/(mg·min)). After integration and applying boundary conditions, q_t_ =0 to q_t_ = q_t_ at t = 0 to t = t, the integrated form of Equation (3) becomes:

t/q_t_ = 1/Kq_e_^2^ + 1/q_e_ * t (4)

where t is the contact time (min), and q_e_ (mg/g) and q^2^ (mg/g) are the amount of solute adsorbed at equilibrium. Figure 4B shows the linear relationship of the graph plot of t/qt *versus* t, from which q_e_ and k can be determined from the slope and intercept, respectively. The linear regression correlation coefficient R_2_^2^ value (0.99) was higher than R^2^_1_ (Table 2). These results confirm that the adsorption data were well represented by the pseudo-second-order kinetic model, this means that the adsorption rate is proportional to the concentration of cadmium (II) squared. As shown in Figure 3, in the beginning, there is high concentration of cadmium ions so the adsorption rate is fast until reach 60 min then the rate become slowly until reach 120 min. Adsorption did not change significantly with further increase in the contact time after 120 min (equilibrium time).

#### 2.3.3. Effect of Adsorbent Dosage

**Figure 5:**
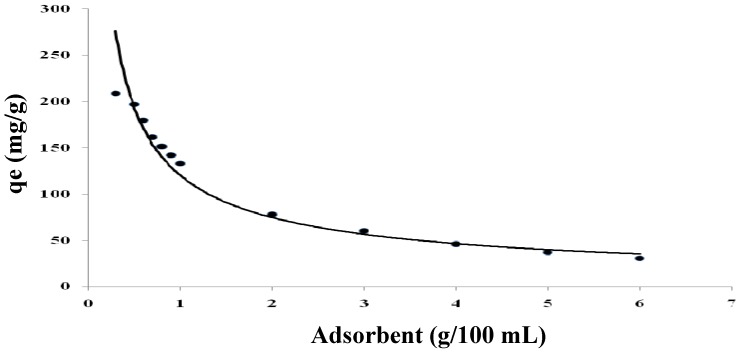
Effect of adsorbent concentration on cadmium(II) biosorption by modified *Tamrix articulata* wastes using 400 mg/L of cadmium at pH 4 and 30 °C.

The amount of cadmium (II) adsorbed varied with adsorbent dosage. As shown in Figure 5, the adsorption capacity of cadmium decreased from 208.7 to 30.6 mg/g with an increase in adsorbent concentration from 0.3 to 6 g/L for an initial cadmium concentration of 400 mg/L. A similar trend was also observed for zinc removal using *Azadirachta indica* as the adsorbent [35].

#### 2.3.4. Adsorption Isotherms

Analysis of isotherm data by fitting different models is an important step in determining a suitable model for design purposes [36].

##### 2.3.4.1. Langmuir Isotherm

The Langmuir equation (5) [37] was applied to the equilibrium data for adsorption of Cd (II) ions onto native and modified *Tamrix articulata* wastes:

C_e_/q_e_ = 1/(q_max_. b) + C_e_/q_max_ (5)

where C_e_ is the equilibrium concentration of the adsorbate (mg/L), q_e_ is the amount of metal ion adsorbed (mg/g), and q_max_ and b are Langmuir constants related to the maximum adsorption capacity (mg/g) and the adsorption energy, respectively. The Langmuir equilibrium constant, K_L_, can be obtained from Equation (6).

K_L_ = q_max_.b. (6)

**Figure 6:**
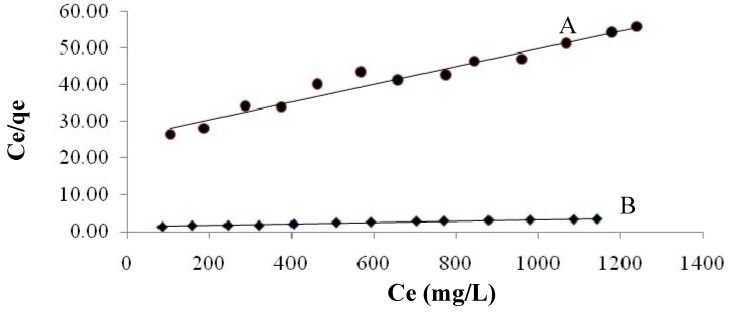
Langmuir biosorption isotherms for cadmium(II) in 0.3 g/L of biosorbent for native (A) and maleic acid-treated *Tamrix articulata* wastes (B).

The linear form of the Langmuir isotherm is shown in Figure 6. The correlation coefficients, R^2^, for the adsorption of Cd(II) ions onto native and maleic acid-treated *Tamrix articulata* wastes are 0.966 and 0.957, respectively, indicating that the adsorption was well fit by the Langmuir isotherm.

##### 2.3.4.2. Freundlich Isotherm

**Figure 7:**
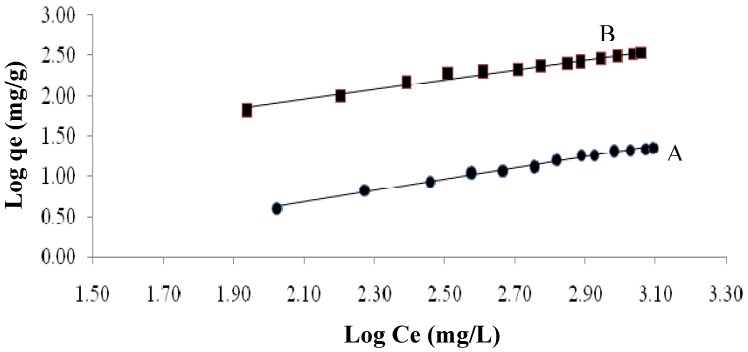
Freundlich biosorption isotherms for cadmium(II) in 0.3 g/L of biosorbent for native (A) and maleic acid-treated *Tamrix articulata* wastes (B).

The Freundlich equation is presented in equation (7):

*Logq_e_ = logK + 1/n logCe* (7)

where Ce is the equilibrium concentration (mg/L) and qe is the amount of metal adsorbed (mg/g) at equilibrium. The quantities K_F_ and n are the Freundlich constants, with K_F_ (mg/g) indicating the adsorbent capacity and n indicating the favorable nature of the process. Plots of log qe *versus* log Ce should be linear, with the slope and intercept of the line obtained corresponding to 1/n and logK_F_, respectively (Figure 7).

The calculated values for the Langmuir and Freundlich isotherm constants are given in Table 3. The adsorption of cadmium (II) onto native and maleic acid-treated *Tamrix articulata* wastes was well correlated with the both the Langmuir and Freundlich equations for the concentration range studied. This means that the adsorption of cadmium (II) onto maleic acid-treated *Tamrix articulata* wastes is favorable. These results are confirmed by the negative ΔGº values (Table 4) which means the spontaneity of the adsorption process.

The linear form of the Langmuir isotherm (Figure 7) indicates that the surface of the maleic acid-treated *Tamrix articulata* wastes is uniform, and all the adsorption sites are equivalent. Also means that adsorbed cadmium molecules do not interact. At the maximum adsorption, only a monolayer is formed: molecules of adsorbate (cadmium) do not deposit on other, already adsorbed, molecules of adsorbate, only on the free surface of the adsorbent.

**Table 3:** Langmuir and Freundlich constants for the adsorption of Cd(II) Ions onto native and maleic acid-treated wastes at pH 4 and 30 °C.

	Constant	Native	Maleic acid-treated plant
Langmuir constants	K_L_	0.024	0.8
	b	0.00057	0.0016
	Q _max._	41.6	500
	R^2^	0.957	0.966
Freundlich constants	K_F_	0.168	4.93
	n	1.44	1.66
	R^2^	0.991	0.973

#### 2.3.5. Thermodynamic Studies

Thermodynamic parameters, such as change in Gibbs free energy (ΔG^o^), enthalpy (ΔH^o^) and entropy (ΔS^o^), were evaluated using Equations (8, 9):

*Log K_d_ = ΔS^o^/2.303R − ΔH^o^/2.303RT* (8)

**Figure 8:**
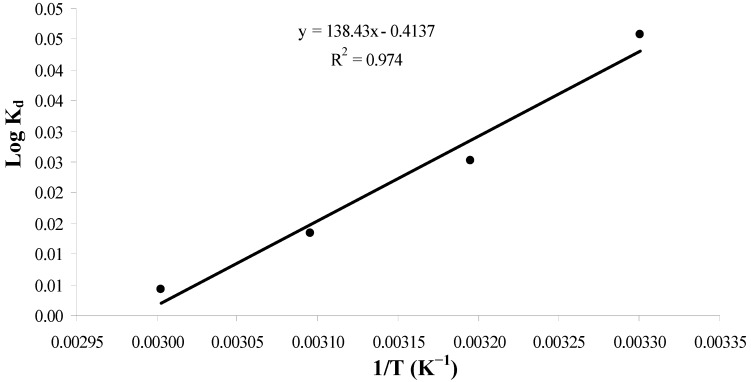
Thermodynamic study of adsorption of Cd(II) onto maleic acid-treated *Tamrix articulata* wastes.

*ΔG^o^ = − RT lnK_d_* (9)

where K_d_ is the equilibrium partition constant calculated as the ratio between sorption capacity (qe) and equilibrium concentration (Ce), R is the gas constant (8.314 J/mol/K) and T is the temperature in Kelvin (K). From Equation (8) a plot of log K_d_ *vs.* 1/T (Figure 8) give ΔH^o^ and ΔS^o^, as shown in Table 4.

The negative ΔHº value indicates the exothermic nature of the process. Moreover, the negative ΔSº value corresponds to a decrease in the degree of freedom of the adsorbed species. The negative ΔGº value confirmed the reaction feasibility and the spontaneity of the adsorption process. Further, the small absolute value for ΔGº obtained in this study indicates that physical adsorption is the predominant mechanism in the sorption process [38].

**Table 4:** Thermodynamic parameters for adsorption of Cd(II) onto maleic acid-treated *Tamrix articulata* wastes.

Temperature T(K)	Thermodynamic parameters		
	ΔG^o^ (kJ/mol)	ΔS^o^ (J/mol/K)	ΔH^o^ (kJ/mol)
303	−0.265	−7.9	−2.6
313	−0.151		
323	−0.082		
333	−0.027		

It is clear from Table 5 that by comparing maximum adsorption capacities (q max) of maleic acid-treated *Tamrix articulata* wastes and other adsorbents from literature, maleic acid-treated *Tamrix articulata* wastes have large capacity in the removal of Cd(II) ions from aqueous solutions. Differences of metal uptake are due to the properties of each adsorbent.

**Table 5:** .Maximum adsorption capacities for cadmium adsorption to different adsorbents.

Adsorbent	q max (mg/g)	References
Techtona grandis L.f	23.20	[39]
Wheat bran	15.71	[40]
Corncorb	55.2	[41]
Juniper fibre	29.54	[42]
Sawdust(Cedrus deodar wood)	73.62	[43]
Spent grain	17.3	[44]
Wheat bran	101	[45]
Algae, marine, dead Biomass	80	[46]
Algae, Nile water	37.43	[47]
Tamrix articulata	195.5	This study

## 3. Experimental

### 3.1. Raw Material and Chemicals

*Tamrix articulate* is a moderately sized tree with feathery foliage and an erect stem usually attaining a height of 40 feet to 50 feet and girth of 5 to 6 feet. It grows faster than any other species in arid tracts. It is distributed in Saudi Arabia in Taif and Riyadh and it can be collected in large amounts each year. *Tamrix articulata* were collected from the Saudi Arabian desert, ground, passed through 200–400 mesh sieves and then washed with hot distilled water until the final effluent was colorless. The resulting material was then dried in an electric oven for 3 h at 60 °C. Maleic acid, cadmium acetate, ethanol and EDTA were all laboratory-grade chemicals supplied by Aldrich (Steinheim, Germany).

### 3.2. Preparation and Characterization of Adsorbent

*Tamrix articulata* wastes were esterified according to the previously described method [48]. A total of 10 g of ground *Tamrix artculata* wastes was placed in a beaker, and maleic acid (0.0043, 0.0022, 0.0011, and 0.0005 mmol/g waste dissolved in the least amount of water) was added under continuous mixing with a mechanical stirrer until a homogeneous paste was obtained. The paste was then transferred to a Pyrex Petri dish and dried for 2 h at 60 °C in an air-circulated oven. The dried material was treated thermally at 140 °C for 2 h, and then cooled to room temperature and ground. Soluble by-products and unreacted acid were removed by extraction in a Soxhlet extractor for 3 h with water-ethanol (20:80) mixture. The final purified material was dried for 2 h at 60 °C. For scanning electron microscopy (SEM) analysis, samples were mounted on an aluminum stub, coated with a thin layer of gold, and then examined using a JEOL microscope (JSM-6380 LA, Japan). Fourier transform infrared (FTIR) spectra of samples were recorded using a Nicolet 6700 FT-IR Spectrometer (Thermo Scientific USA). Elemental analysis of native and maleic acid-treated *Tamrix articulata* wastes was performed using Series II CHN analyzer (Perkin Elmer, USA).

### 3.3. Carboxyl Content

**Scheme 1:**
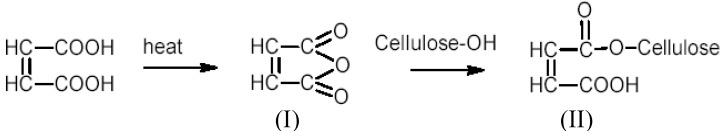
Reaction of cellulose with maleic acid.

The carboxyl content of the native and modified *Tamrix articulata* wastes was determined according to the method reported in [49]. Previous studies [50,51] have indicated that carboxylic acids can form anhydrides (I) when subjected to heat treatment (Scheme 1). The presence of maleic acid and cellulosic material in the *Tamrix articulata* wastes during the heating process would allow an anhydride (I) to react with the hydroxyl groups of the cellulose in the *Tamrix articulata* wastes, forming a cellulose maleate adduct (II) [26].

**Figure 9:**
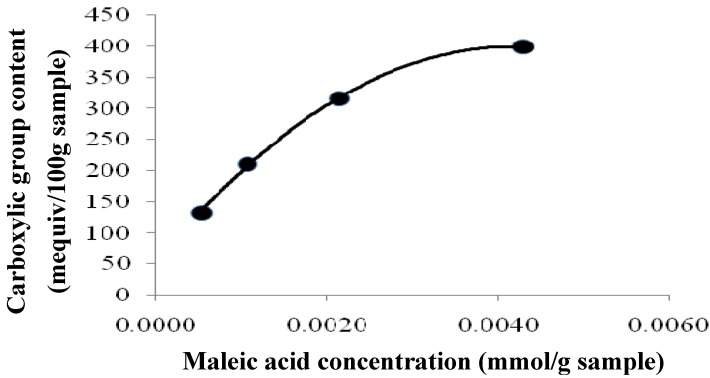
Effect of maleic acid concentration on the carboxyl group content of esterified *Tamrix articulata* wastes.

As shown in Figure 9, the influence of the maleic acid concentration [mmol/g (*Tamrix articulata* wastes)] on the extent of esterification as expressed in mequiv COOH/100 g sample. The extent of esterification increased significantly as the maleic acid concentration was increased, within the range studied. This enhancement in the ester group content could be interpreted in terms of the greater availability of maleic acid molecules to be converted to anhydride (I).

### 3.4. Adsorption Studies

Known volumes (100 mL) of each cadmium (II) ion solution [100-1,250 mg/L] were placed in 125-mL Erlenmeyer flasks containing 0.03 g of adsorbent. The contents were shaken at 150 rpm for 2 h in a thermostatic shaking water bath at 30 °C. At the end of 2 h, the metal ion solutions were separated by filtration. Blank experiments without added adsorbents were performed at the same time. The metal ion concentrations were determined by direct detection against standard EDTA solutions before and after adsorption.

## 4. Conclusions

The maleic acid-treated *Tamrix articulata* wastes investigated in this study showed good potential for the removal of cadmium (II) from aqueous solutions. The maximum adsorption capacity (qe) occurred at pH 4 and a contact time of 2 h. The adsorption isotherms were well fit by the Langmuir and Freundlich equations. The biosorption process was best described by a pseudo-second-order equation. Thermodynamic parameters (ΔG^o^, ΔH^o^ and ΔS^o^) showed that the adsorption process is spontaneous and exothermic in nature. Further future work will be comparative study to test the treated *Tamrix articulata* wastes in adsorption of other toxic metals and also in adsorption of some organic pollutants from water.
